# Women’s barriers for contacting their general practitioner when bothered by urinary incontinence: a population-based cross-sectional study

**DOI:** 10.1186/s12894-021-00864-x

**Published:** 2021-07-12

**Authors:** Dorte Ejg Jarbøl, Peter Fentz Haastrup, Sanne Rasmussen, Jens Søndergaaard, Kirubakaran Balasubramaniam

**Affiliations:** Research Unit of General Practice, Department of Public Health, University of Southern Denmark, J.B. Winsløws Vej 9A, 2. Sal, 5000 Odense C, Denmark

**Keywords:** Urinary incontinence, Healthcare-seeking, Socioeconomic characteristics, Barriers

## Abstract

**Background:**

Urinary incontinence (UI) is a frequently occurring condition among women and increases with age. Effective treatments exist but many women hesitate to contact their general practitioner (GP) regarding UI. Therefore, it is important to generate knowledge regarding barriers for healthcare-seeking. Several factors such as age, duration and number of symptoms are associated with healthcare-seeking. How socioeconomic status (SES) is associated with experiencing barriers for healthcare-seeking for UI has not been explored. The objectives of this study were to: (1) analyze frequencies of barriers for healthcare-seeking, and (2) investigate associations between SES and barriers for contacting the GP, among women reporting bothersome UI.

**Method:**

A cross-sectional web-based questionnaire study of symptoms occurrence among 51,090 randomly selected women. This study investigates reported symptoms of three types of UI (stress UI, urge UI and UI without stress or urge) and reported barriers for GP contact combined with register data on SES.

**Results:**

A total of 4,051 (16.4%) women reported to be bothered by either stress UI (9.1%), urge UI (4.0%) or incontinence without stress or urge (2.4%) and 76.3%, 70%, and 64% respectively, had not contacted their GP regarding the symptom(s). The most frequently reported barriers were ‘being too embarrassed’ (19.3%) and ‘being too busy’ (18.4%) for stress incontinence, and ‘being too embarrassed (19.0%) or ‘worried about wasting the doctor’s time’ (16.9%) for women with bothersome urge UI or UI without stress or urge. Younger women had higher odds of reporting barriers and the barriers embarrassment and being worried about what the doctor might find were significantly associated with lower educational level.

**Conclusion:**

Women with lower educational level have an increased risk of not seeking healthcare for UI symptoms. The GP should be aware of identifying women bothered by UI for whom effective treatment options to alleviate the symptoms are available.

**Supplementary Information:**

The online version contains supplementary material available at 10.1186/s12894-021-00864-x.

## Introduction

Urinary incontinence (UI) is frequent among women worldwide [1]. UI may be classified due to the nature of the symptom, such as stress incontinence, urge incontinence and mixed incontinence [2]. The prevalence varies substantially among different study populations and is highly age dependent [3–5]. It has been estimated that 20% of Danish women in the age group 40–60 years and 44% in the age group 80+ years report UI [6]. Not only is UI common, it has also been shown to have a negative impact on daily activity and health related quality of life [7].

There are several approaches to alleviate UI including both non-pharmacological, pharmacological and surgical treatment strategies [8]. A prerequisite for treatment is, however, that women experiencing UI seek healthcare. A review of five studies showed that less than 38% of women reporting UI contacted their general practitioner (GP) [9]. The decision of whether to contact the GP may be affected by numerous factors where some act as barriers and others as drivers for healthcare-seeking. Previous studies have found that healthcare-seeking with UI is strongly associated with age, the duration of symptoms, the number of symptoms and the symptoms being worrying or influencing the everyday life [6]. Other studies have demonstrated that embarrassment and negative attitudes towards healthcare use are negatively associated with healthcare-seeking for UI [10]. Further, healthcare-seeking for intimate symptoms varies between socioeconomic groups [11]. To facilitate diagnose and treatment for women bothered by UI but hesitating to seek healthcare it is important to generate knowledge regarding barriers for healthcare-seeking. How socioeconomic status (SES) is associated with experiencing barriers for healthcare-seeking for UI has not yet been explored.

Hence, the aim of this study was to analyze (1) frequencies of barriers for healthcare-seeking, and (2) the associations between commonly described barriers towards GP contact and SES among women bothered by UI.

## Material and methods

This study is part of a nationwide cross-sectional cohort study, the Danish Symptom Cohort (DaSC). DaSC was designed as a web-based questionnaire study to which 100 000 individuals aged 20 years or above were randomly selected from the Danish Civil Registration System (CRS). The CRS contains information about date of birth, gender and a unique identification number for every Danish resident [12], which is used as the key identifier in all health and social registers. The selected individuals received a letter explaining the purpose of the study. The letter included a login for a secure web page with the questionnaire. Those without internet access were able to complete the questionnaire as a telephone interview. Individuals who did not respond within two weeks received a reminder letter, and after additional two weeks non-responders were contacted by telephone by a private marketing company. During this reminder procedure, reasons for non-participation were assessed when possible. Data were collected from June to December 2012. Details about the study sample and validity of the questionnaire are reported in Rasmussen et al. [13].

### Questionnaire

The questionnaire was covering 44 predefined symptoms regarding a wide area of clinically relevant symptoms. When the respondents confirmed a symptom experience, a number of follow-up questions were asked concerning the onset of the symptom, the symptom’s influence on daily activities, concerns about the symptom, whether the respondent had consulted the GP regarding the symptom and considerations about contacting the GP with the symptom in question.

The reporting of three types of UI (stress UI, urge UI and UI without stress or urge) form the basis of this paper. *The questions were phrased;* Have you within the last 4 weeks experienced any of the these? *That the urge to urinate is so strong that you cannot make it to the toilet in time (Urge UI), Involuntary urination during exertion, e.g. coughing, sneezing, lifting and exercise (Stress UI), and Involuntary urination (incontinence) without effort and urge (leakage).* To report to what extent, they were concerned about UI and to what extent UI interfered with their usual daily activities, five-point Likert scale with the options: “not at all”, “slightly”, “moderate”, “quite a bit” and “extremely”, was used. Respondents who were either moderately to extremely concerned and/or moderately to extremely influenced in their daily activities due to UI were considered bothered by UI, in the following described as bothersome UI.

This paper focuses on the women who had not contacted the GP with bothersome UI and the possible barriers for contacting the GP. Four common barriers for healthcare-seeking were included based on a literature search [14]. For each reported symptom without GP contact, the question was phrased: “You have not been in contact with your general practitioner regarding the symptom or discomfort you have experienced; did you have any of the following considerations about contacting your general practitioner?” The following options were given: “I would be too embarrassed”, “I would be worried about wasting the doctor’s time”, “I would be worried about what the doctor might find”, and “I was too busy to make time to go to the doctor” (Each of the questions could be answered with “yes” or”no”). Further, an “other” option was given, which was a free text box, where the respondents could elaborate on other options than the abovementioned. The free text box answers were not included in the present study.

The methodological framework for developing, and pilot- and field-testing the questionnaire is described in detail elsewhere [13].

### Register data

For all respondents register data regarding SES (educational level, household income, cohabitation status, ethnicity and labor market affiliation) were collected from Statistics Denmark using the unique identification number in CRS enabling linkage between registers [15–17].

### Statistical analyses

The study includes women reporting UI with the primary focus on women who reported bothersome UI and further reported, that they had not contacted their GP regarding the symptom(s). The term bothersome covers UI which was reported as either moderately to extremely concerning and/or moderately to extremely influencing on daily activities.

The proportions of women with bothersome UI and with no GP contact, respectively, are presented with the distribution of each covariate. The proportions of reported barriers among women with no GP contact with bothersome UI are presented for each type of UI. Logistic regression models were used to calculate unadjusted and adjusted odds ratios (ORs) for associations between each reported barrier towards GP contact among women who did not contact the GP and each of the covariates for the separate types of bothersome UI. The variables considered for analyses were age group, educational level, income, labor market affiliation, cohabitation status and ethnicity. Because of too few women reporting bothersome urge UI and UI without stress or urge we were not allowed to perform separate analyses of the associations between SES and barriers for GP contact for these respondents due to Danish data protection legislation. Hence, the two categories were merged in these analyses.

Age was categorized as 20–39, 40–59, 60–79 or 80+ years old. Education was categorized according to the highest attained educational level: low (< 10 years, i.e. primary and lower secondary school); middle (10–14 years, i.e. vocational education, upper secondary school and shorter education); or high (≥ 15 years, i.e. medium- or long-term higher education). Average disposable income was defined as the entire household income after taxation, adjusted for number of persons in the household in the year of filling in the questionnaire. Disposable income was categorized as low income (1st quartile), middle income (2nd and 3rd quartiles) or high income (4th quartile). Labor market affiliation was categorized as working, retirement pension, disability pension or out of the workforce according to the status each respondent predominantly had in the year of filling in the questionnaire. Cohabitation status at the time of participating in the survey was categorized as cohabiting/married or single. Ethnicity was categorized as people of Danish origin, immigrants, or descendants of immigrants.

All statistical tests used a significance level of p < 0.05. Data analyses were conducted using STATA statistical software 13.1 (StataCorp, College Station, TX, USA).

## Results

A total of 51,090 Danish women were invited to participate in the survey. Among those 24,624 (48.2%) did not respond to the questionnaire or were ineligible for the study since they had either died, could not be reached due to unknown addresses, had severe illness (including dementia), had language problems, or had moved abroad. Among the 26,466 respondents 24,683 (93.3%) had complete data regarding the survey and register data. 

A total of 4051 (16.4%) women reported to be bothered by either stress UI (9.1%), urge UI (4.0%) or incontinence without stress or urge (2.4%). Some 70% of those bothered by stress UI had not contacted their GP regarding the symptom. Similarly, 76.3% and 64% had not contacted their GP regarding bothersome urge UI and bothersome UI without stress or urge, respectively, Table 1.

**Table 1:** Socioeconomic characteristics for women with bothersome* urinary incontinence (UI) and with no GP contact

	Total N	Stress UI		Urge UI		UI without stress or urge	
		N (%)	No GP contact (%)	N (%)	No GP contact (%)	N (%)	No GP contact (%)
All	24,683	2246 (9.1)	1713 (76.3)	1211 (4.9)	848 (70.0)	594 (2.4)	380 (64.0)
*Age*							
20–39	5554	281 (5.1)	224 (79.7)	126 (2.3)	89 (70.6)	46 (0.8)	30 (65.2)
40–59	10,680	1012 (9.5)	811 (80.1)	465 (4.4)	356 (76.6)	206 (1.9)	158 (76.7)
60–79	7732	861 (11.1)	617 (71.7)	543 (7.0)	354 (65.2)	293 (3.8)	165 (56.3)
80+	717	92 (12.8)	61 (66.3)	77 (10.7)	49 (63.6)	49 (6.8)	27 (55.1)
*Marital status*							
Single	6326	579 (9.2)	420 (72.5)	375 (5.9)	247 (65.9)	205 (3.2)	122 (59.5)
Married/Cohabiting	18,357	1667 (9.1)	1293 (77.6)	836 (4.6)	601 (71.9)	389 (2.1)	258 (66.3)
*Educational level*							
Low (< 10 years)	3069	403 (13.1)	278 (69.0)	263 (8.6)	171 (65.0)	135 (4.4)	72 (53.3)
Middle (10–14 years)	12,644	1196 (9.5)	922 (77.1)	609 (4.8)	437 (71.8)	306 (2.4)	204 (66.7)
High (> = 15 years)	8970	647 (7.2)	513 (79.3)	339 (3.8)	240 (70.8)	153 (1.7)	104 (68.0)
*Labor market affiliation*							
Working	16,429	1263 (7.7)	1022 (80.9)	529 (3.2)	398 (75.2)	235 (1.4)	172 (73.2)
Pension	5820	682 (11.7)	480 (70.4)	470 (8.1)	307 (65.3)	267 (4.6)	148 (55.4)
Out of workforce	1360	129 (9.5)	99 (76.7)	90 (6.6)	69 (76.7)	35 (2.6)	27 (77.1)
Disability pension	1074	172 (16.0)	112 (65.1)	122 (11.4)	74 (60.7)	57 (5.3)	33 (57.9)
*Equivalence weighted disposable income*							
Low (1st quartile)	4189	425 (10.1)	301 (70.8)	277 (6.6)	180 (65.0)	140 (3.3)	82 (58.6)
Middle (2nd and 3rd quartile)	13,004	1217 (9.4)	928 (76.3)	649 (5.0)	455 (70.1)	320 (2.5)	201 (62.8)
High (4th quartile)	7490	604 (8.1)	484 (80.1)	285 (3.8)	213 (74.7)	134 (1.8)	97 (72.4)
*Ethnicity*							
Danish	23,155	2130 (9.2)	1628 (76.4)	1149 (5.0)	808 (70.3)	567 (2.4)	364 (64.2)
Immigrants/descendants of immigrants	1528	116 (7.6)	85 (73.3)	62 (4.1)	40 (64.5)	27 (1.8)	16 (59.3)

*Bothersome UI is defined by being either moderately to extremely concerned and/or moderately to extremely influenced in their daily activities due to UI

In the analyses of associations between not contacting the GP regarding each UI subtype and SES we found that women with high educational level had higher odds of no GP contact with stress UI (OR = 1.42, 95% CI 1.05–1.93). Women on disability pension had lower odds of reporting no GP contact regarding both stress UI (OR = 0.50, 95% CI 0.35–0.72) and urge UI (OR = 0.53, 95% CI 0.34–0.81) (Table 2).

**Table 2:** Associations between not contacting the GP and SES regarding women reporting bothersome urinary incontinence (UI)

	Stress UI		Urge UI		UI without stress or urge	
	Crude OR (95% CI)	Adj OR (95% CI)	Crude OR (95% CI)	Adj OR (95% CI)	Crude OR (95% CI)	Adj OR (95% CI)
*Age*						
20–39	1	1	1	1	1	1
40–59	1.03 (0.74–1.43)	1.06 (0.76–1.48)	1.36 (0.88–2.11)	1.35 (0.87–2.10)	1.76 (0.88–3.49)	1.77 (0.89–3.53)
60–79	0.64 (0.46–0.89)	0.72 (0.51–1.01)	0.78 (0.51–1.19)	0.81 (0.53–1.25)	0.69 (0.36–1.32)	0.77 (0.40–1.49)
80+	0.50 (0.30–0.84)	0.62 (0.36–1.07)	0.73 (0.40–1.33)	0.81 (0.44–1.50)	0.65 (0.29–1.50)	0.75 (0.32–1.76)
*Marital status*						
Single	1	1	1	1	1	1
Married/Cohabiting	1.31 (1.05–1.62)	1.18 (0.94–1.47)	1.33 (1.02–1.72)	1.27 (0.97–1.66)	1.34 (0.94–1.90)	1.17 (0.81–1.70)
*Educational level*						
Low (< 10 years)	1	1	1	1	1	1
Middle (10–14 years)	1.51 (1.18–1.94)	**1.32** (**1.02–1.71)**	1.37 (1.00–1.86)	1.21 (0.88–1.66)	1.75 (1.16–2.65)	1.42 (0.92–2.18)
High (> = 15 years)	1.72 (1.30–2.29)	**1.42** (**1.05–1.93**)	1.30 (0.92–1.84)	1.14 (0.80–1.63)	1.86 (1.15–3.00)	1.47 (0.89–2.42)
*Labor market affiliation*						
Working	1	1	1	1	1	1
Retirement pension	0.56 (0.45–0.70)	0.77 (0.54–1.09)	0.62 (0.47–0.82)	0.94 (0.62–1.43)	0.46 (0.31–0.66)	0.77 (0.43–1.38)
Out of workforce	0.78 (0.51–1.20)	0.82 (0.53–1.27)	1.08 (0.64–1.83)	1.10 (0.65–1.88)	1.24 (0.53–2.86)	1.60 (0.66–3.89)
Disability pension	0.44 (0.31–0.62)	**0.50** (**0.35–0.72)**	0.51 (0.34–0.77)	**0.53** (**0.34–0.81**)	0.50 (0.28–0.92)	0.57 (0.30–1.08)
*Equivalence weighted disposable income*						
Low (1st quartile)	1	1	1	1	1	1
Middle (2nd and 3rd quartile)	1.32 (1.03–1.69)	1.12 (0.86–1.46)	1.26 (0.94–1.70)	1.11 (0.81–1.53)	1.19 (0.80–1.79)	0.97 (0.63–1.51)
High (4th quartile)	1.66 (1.24–2.22)	1.30 (0.94–1.80)	1.59 (1.11–2.29)	1.29 (0.85–1.95)	1.85 (1.12–3.08)	1.24 (0.70–2.21)
*Ethnicity*						
Danish	1	1	1	1	1	1
Immigrants/ descendants of immigrants	0.85 (0.55–1.29)	0.81 (0.53–1.25)	0.77 (0.45–1.31)	0.70 (0.41–1.21)	0.81 (0.37–1.78)	0.78 (0.35–1.76)

Bold indicates a p value below 0.05

A total of 64.6% of the women with bothersome stress incontinence and no GP contact reported barriers for GP contact. The most frequent predefined barriers were ‘being too embarrassed’ and ‘being too busy’ reported by 19.3% and 18.4%, respectively (See Additional file 1: Table 1A).

Among women with bothersome urge incontinence or incontinence without stress or urge a total of 61.2% of the women with no GP contact reported at least one barrier towards GP contact. Most women reported ‘being too embarrassed (19.0%) or ‘worried about wasting the doctor’s time’ (16.9%). (See Additional file 1: Table 2A).

In total, 27% of the women with bothersome UI reported ‘other’ barriers for both symptom categories, indicating that the women had other barriers than the predefined barriers (See Additional file 1: Table 1A and 2A).

In the analyses of associations between age, SES and reported barriers for GP contact regarding stress UI we found that high age was associated with lower odds of reporting all four barriers for GP contact. Further, high income and high educational level were associated with lower odds of reporting embarrassment and being worried about what the doctor might find but higher odds for choosing the “other” category as barrier. Women being outside the workforce had lower odds of being too busy to go to the GP whereas non-Danish ethnicity was associated with higher odds of being too busy (Table 3).

**Table 3:** Associations between age, SES and reported barriers for GP contact regarding stress UI among women with no GP contact

	Being too embarrassed		Wasting the GP's time		Worried about what the GP might find	
	Crude OR (95% CI)	Adj. OR* (95% CI)	Crude OR (95% CI)	Adj. OR* (95% CI)	Crude OR (95% CI)	Adj. OR* (95% CI)
*Age*						
20–39	1	1	1	1	1	1
40–59	0.72 (0.51–1.01)	**0.68 ** (**0.48 0.96)**	0.61 (0.43–0.86)	**0.58 ** (**0.41–0.83)**	0.74 (0.46–1.19)	0.66 (0.41–1.07)
60–79	0.46 (0.32–0.67)	**0.41** (**0.28–0.61)**	0.49 (0.34–0.72)	**0.48** (**0.32–0.70)**	0.49 (0.29–0.82)	**0.39** (**0.22–0.68)**
80+	0.34 (0.15–0.79)	**0.31** (**0.13–0.73)**	0.38 (0.16–0.87)	**0.37** (**0.15–0.87)**	0.98 (0.40–2.37)	**0.78** (**0.31–2.00)**
*Marital status*						
Single	1	1	1	1	1	1
Married/Cohabiting	1.21 (0.91–1.62)	1.09 (0.81–1.47)	1.11 (0.82–1.50)	1.02 (0.75–1.38)	1.18 (0.78–1.78)	1.19 (0.77–1.84)
*Educational leve*						
Low (< 10 years)	1	1	1	1	1	1
Middle (10–14 years)	1.02 (0.73–1.42)	0.82 (0.57–1.16)	1.36 (0.94–1.98)	1.19 (0.81–1.76)	0.91 (0.58–1.42)	0.75 (0.47–1.21)
High (> = 15 years)	0.90 (0.62–1.30)	**0.65** (**0.44–0.96)**	1.14 (0.75–1.71)	0.91 (0.59–1.40)	0.56 (0.32–0.95)	**0.42** (**0.23–0.74)**
*Labor market affiliation*						
Working	1	1	1	1	1	1
Retirement pension	0.64 (0.48–0.86)	1.00 (0.61–1.66)	0.70 (0.52–0.95)	0.87 (0.53–1.43)	0.83 (0.55–1.26)	1.20 (0.55–2.61)
Out of workforce	1.39 (0.87–2.24)	1.33 (0.82–2.16)	1.44 (0.88–2.36)	1.42 (0.86–2.35)	1.74 (0.93–3.25)	1.60 (0.85–3.03)
Disability pension	0.58 (0.33–1.01)	0.60 (0.34–1.08)	0.81 (0.47–1.39)	0.91 (0.52–1.58)	0.96 (0.47–1.97)	0.98 (0.47–2.07)
*Equivalence weighted disposable income*						
Low (1st quartile)	1	1	1	1	1	1
Middle (2nd and 3rd quartile)	0.96 (0.70–1.32)	0.85 (0.61–1.20)	1.15 (0.81–1.63)	1.08 (0.75–1.58)	0.58 (0.38–0.88)	**0.53** (**0.34–0.83)**
High (4th quartile)	0.65 (0.45–0.94)	**0.58** (**0.38–0.88)**	0.97 (0.65–1.44)	0.96 (0.62–1.49)	0.41 (0.25–0.68)	**0.38** (**0.22–0.68)**
*Ethnicity*						
Danish	1	1	1	1	1	1
Immigrants and descendants of immigrants	1.40 (0.84–2.33)	1.31 (0.78–2.20)	1.23 (0.71–2.14)	1.17 (0.67–2.04)	0.99 (0.45–2.20)	0.91 (0.41–2.03)

	Being too busy		Other		None	
	Crude OR (95% CI)	Adj. OR* (95% CI)	Crude OR (95% CI)	Adj. OR* (95% CI)	Crude OR (95% CI)	Adj. OR* (95% CI)
*Age*						
20–39	1	1	1	1	1	1
40–59	0.89 (0.63–1.26)	0.90 (0.63–1.27)	0.97 (0.70–1.34)	1.17 (0.84–1.64)	1.17 (0.84–1.63)	1.07 (0.76–1.50)
60–79	0.37 (0.25–0.54)	**0.37** (**0.25–0.56)**	0.78 (0.55–1.09)	1.15 (0.80–1.65)	2.35 (1.67–3.29)	**1.91** (**1.35–2.71)**
80+	0.38 (0.17–0.89)	**0.38** (**0.16–0.91)**	0.41 (0.19–0.89)	0.69 (0.31–1.54)	3.85 (2.14–6.94)	**2.83** (**1.53–5.23)**
*Marital status*						
Single	1	1	1	1	1	1
Married/Cohabiting	1.05 (0.79–1.40)	0.87 (0.65–1.18)	1.17 (0.91–1.51)	1.03 (0.79–1.35)	0.74 (0.59–0.93)	0.90 (0.71–1.14)
*Educational leve*						
Low (< 10 years)	1	1	1	1	1	1
Middle (10–14 years)	1.61 (1.09–2.37)	1.24 (0.83–1.85)	2.12 (1.44–3.12)	**2.08** (**1.41–3.09)**	0.55 (0.42–0.72)	**0.68** (**0.51–0.90)**
High (> = 15 years)	1.71 (1.14–2.59)	1.20 (0.78–1.86)	4.77 (3.21–7.09)	**4.73** (**3.14–7.13)**	0.36 (0.26–0.48)	**0.47** (**0.34–0.65)**
*Labor market affiliation*						
Working	1	1	1	1	1	1
Retirement pension	0.27 (0.19–0.39)	**0.32** (**0.19–0.54)**	0.61 (0.47–0.79)	0.78 (0.52–1.18)	2.64 (2.11–3.31)	**1.77** (**1.23–2.56)**
Out of workforce	0.52 (0.29–0.94)	**0.50** (**0.28–0.90)**	0.58 (0.35–0.97)	0.70 (0.42–1.18)	1.30 (0.84–2.02)	1.17 (0.75–1.83)
Disability pension	0.20 (0.09–0.44)	**0.20** (**0.09–0.44)**	0.80 (0.52–1.25)	1.05 (0.66–1.69)	1.88 (1.26–2.80)	**1.54** (**1.01–2.33)**
*Equivalence weighted disposable income*						
Low (1st quartile)	1	1	1	1	1	1
Middle (2nd and 3rd quartile)	1.03 (0.73–1.46)	0.90 (0.62–1.32)	1.39 (1.01–1.91)	1.03 (0.73–1.44)	0.84 (0.64–1.10)	1.14 (0.85–1.52)
High (4th quartile)	1.40 (0.96–2.03)	1.25 (0.82–1.90)	1.89 (1.35–2.65)	1.19 (0.82–1.75)	0.63 (0.47–0.86)	0.96 (0.68–1.34)
*Ethnicity*						
Danish	1	1	1	1	1	1
Immigrants and descendants of immigrants	1.81 (1.11–2.95)	**1.68** (**1.02–2.77)**	0.77 (0.46–1.31)	0.74 (0.43–1.26)	0.80 (0.50–1.28)	0.85 (0.53–1.38)

*Adjustments were made for all other covariates

Bold indicates a p value below 0.05

When analyzing the associations between age, SES and reported barriers for GP contact regarding urge UI or UI without stress or urge we found that high age was associated with lower odds of being worried about wasting the GP’s time and being too busy to see the GP. Women with high educational level had lower odds of reporting being embarrassed, being worried about what the doctor might find and being worried about wasting the GP’s time. Women being outside the workforce had lower odds of being too busy. Cohabiting was associated with higher odds of being worried about wasting the GP’s time (Table 4).

**Table 4:** Associations between age, SES and reported barriers for GP contact regarding urge UI or UI without stress or urge among women with no GP contact

	Being too embarrassed		Wasting the GP's time		Worried about what the GP might find	
	Crude OR (95% CI)	Adj. OR* (95% CI)	Crude OR (95% CI)	Adj. OR* (95% CI)	Crude OR (95% CI)	Adj. OR* (95% CI)
*Age*						
20–39	1	1	1	1	1	1
40–59	1.20 (0.73–2.00)	1.16 (0.70–1.93)	0.62 (0.38–1.02)	**0.58** (**0.35–0.97)**	0.74 (0.40–1.39)	0.70 (0.38–1.32)
60–79	0.66 (0.39–1.11)	0.59 (0.35–1.02)	0.57 (0.35–0.95)	**0.52** (**0.31–0.87)**	0.69 (0.37–1.29)	0.59 (0.31–1.12)
80+	0.56 (0.23–1.33)	0.54 (0.22–1.31)	0.59 (0.26–1.31)	0.59 (0.26–1.34)	0.43 (0.14–1.37)	0.38 (0.12–1.23)
*Marital status*						
Single	1	1	1	1	1	1
Married/Cohabiting	1.40 (0.99–2.00)	1.32 (0.92–1.88)	1.47 (1.01–2.13)	**1.48** (**1.01–2.17)**	1.11 (0.71–1.73)	1.09 (0.69–1.70)
*Educational level*						
Low (< 10 years)	1	1	1	1	1	1
Middle (10–14 years)	0.84 (0.57–1.25)	0.69 (0.46–1.04)	0.93 (0.62–1.40)	0.85 (0.56–1.29)	0.75 (0.46–1.21)	0.68 (0.41–1.12)
High (> = 15 years)	0.74 (0.48–1.16)	**0.60** (**0.37–0.95)**	0.60 (0.37–0.97)	**0.53** (**0.32–0.87)**	0.50 (0.28–0.90)	**0.44** (**0.24–0.81)**
*Labor market affiliation*						
Working	1	1	1	1	1	1
Retirement pension	0.63 (0.44–0.89)	1.06 (0.57–1.97)	0.90 (0.63–1.28)	1.06 (0.58–1.93)	0.91 (0.57–1.45)	0.87 (0.42–1.78)
Out of workforce	1.05 (0.60–1.84)	1.00 (0.56–1.78)	1.10 (0.60–2.01)	1.02 (0.55–1.90)	2.20 (1.17–4.15)	2.00 (1.04–3.83)
Disability pension	1.02 (0.59–1.74)	0.99 (0.56–1.73)	1.17 (0.67–2.06)	1.21 (0.68–2.18)	1.59 (0.83–3.08)	1.47 (0.74–2.90)
*Equivalence weighted disposable income*						
Low (1st quartile)	1	1	1	1	1	1
Middle (2nd and 3rd quartile)	0.90 (0.61–1.33)	0.82 (0.54–1.24)	1.10 (0.74–1.65)	1.08 (0.70–1.66)	0.74 (0.46–1.17)	0.74 (0.45–1.21)
High (4th quartile)	0.89 (0.57–1.38)	0.76 (0.46–1.25)	0.70 (0.42–1.14)	0.70 (0.40–1.22)	0.44 (0.24–0.81)	**0.45** (**0.23–0.89)**
*Ethnicity*						
Danish	1	1	1	1	1	1
Immigrants and descendants of immigrants	1.44 (0.75–2.75)	1.32 (0.68–2.55)	1.51 (0.77–2.93)	1.49 (0.76–2.94)	1.65 (0.76–3.60)	1.60 (0.72–3.53)

	Being too busy		Other		None	
	Crude OR (95% CI)	Adj. OR* (95% CI)	Crude OR (95% CI)	Adj. OR* (95% CI)	Crude OR (95% CI)	Adj. OR* (95% CI)
*Age*						
20–39	1	1	1	1	1	1
40–59	0.74 (0.45–1.22)	0.76 (0.46–1.24)	1.47 (0.92–2.37)	1.62 (1.00–2.63)	0.94 (0.60–1.47)	0.90 (0.57–1.42)
60–79	0.35 (0.21–0.60)	**0.36** (**0.21–0.63)**	1.01 (0.62–1.64)	1.24 (0.75–2.04)	2.13 (1.36–3.32)	**1.97** (**1.25–3.09)**
80+	0.39(0.16–0.95)	**0.39** (**0.16–0.96)**	0.81 (0.38–1.71)	0.90 (0.42–1.94)	2.49 (1.32–4.72)	**2.35** (**1.23–4.50)**
*Marital status*						
Single	1	1	1	1	1	1
Married/Cohabiting	0.96 (0.67–1.38)	0.87 (0.60–1.25)	0.87 (0.65–1.16)	0.79 (0.58–1.07)	0.91 (0.70–1.19)	1.03 (0.78–1.37)
*Educational level*						
Low (< 10 years)	1	1	1	1	1	1
Middle (10–14 years)	1.41 (0.87–2.29)	1.15 (0.70–1.89)	1.73 (1.14–2.62)	**1.67** (**1.09–2.55)**	0.73 (0.53–1.01)	0.89 (0.64–1.25)
High (> = 15 years)	1.65 (0.98–2.77)	1.29 (0.76–2.21)	3.55 (2.29–5.50)	**3.48** (**2.22–5.44)**	0.48 (0.33–0.70)	**0.60** (**0.41–0.88)**
*Labor market affiliation*						
Working	1	1	1	1	1	1
Retirement pension	0.23 (0.14–0.35)	**0.17** (**0.09–0.33)**	0.59 (0.43–0.80)	0.62 (0.38–1.00)	2.52 (1.91–3.32)	1.75 (1.12–2.73)
Out of workforce	0.34 (0.16–0.73)	**0.31** (**0.14–0.67)**	0.84 (0.50–1.42)	0.96 (0.56–1.64)	0.98 (0.59–1.64)	0.96 (0.57–1.62)
Disability pension	0.26 (0.12–0.57)	**0.25** (**0.11–0.55)**	0.75 (0.46–1.24)	0.86 (0.51–1.44)	1.57 (0.99–2.48)	1.43 (0.89–2.29)
*Equivalence weighted disposable income*						
Low (1st quartile)	1	1	1	1	1	1
Middle (2nd and 3rd quartile)	1.10 (0.71–1.71)	1.05 (0.65–1.69)	1.46 (1.00–2.13)	1.29 (0.87–1.93)	0.81 (0.60–1.11)	0.94 (0.67–1.32)
High (4th quartile)	1.39 (0.85–2.26)	1.33 (0.76–2.32)	2.39 (1.58–3.60)	**1.89** (**1.19–3.00)**	0.65 (0.45–0.93)	0.84 (0.56–1.27)
*Ethnicity*						
Danish	1	1	1	1	1	1
Immigrants and descendants of immigrants	1.68 (0.86–3.28)	1.54 (0.78–3.03)	0.97 (0.52–1.82)	0.89 (0.46–1.69)	0.69 (0.38–1.26)	0.80 (0.43–1.47)

*Adjustments were made for all other covariates

Bold indicates a p value below 0.05

## Discussion

### Main findings

We found that bothersome UI is common among women in the general population. Most women bothered by UI had not contacted their GP regarding their incontinence symptoms. Younger women had higher odds of reporting barriers and the barriers embarrassment and being worried about what the doctor might find were significantly associated with lower educational level.

### Strengths and limitations

A major strength of this study is that we included a large number of randomly selected women (51,090 women) and had a relatively high response rate (54.5% among women) comparable to other studies of UI in the general population [5]. We obtained SES information from high-quality registers with a high degree of coverage and accuracy [15–17] compared to self-reported SES which may be subject to reporting bias. An overall responder analysis of the entire study cohort including both men and women showed that respondents were more often cohabiting, had higher educational level, had higher income, were of Danish origin and were more often working [18].

Our design using a web-based questionnaire is an advantage as it provides anonymity and thereby may enhance response rate from women not wishing to talk about UI. Moreover, the survey explored a range of different symptoms, and thereby trying to avoid women with UI to be more prone to answer questionnaire. The understanding and interpretation of the questions regarding UI might depend on SES, e.g. age and educational level. However, the field and pilot testing did not reveal problems in relation to this. Participants were asked to recall symptom experience during the preceding four weeks. The short recall period reduces risk of recall error. As we addressed bothersome UI i.e. which either worried or influenced daily activities, it seems reasonable to assume a correct recall within this time frame.

A minority of the respondents (2.4%) responded to the questionnaire via telephone interview. It is possible that information from the interviews are different from the information derived from the online version. The difference was sought minimised by using trained interviewers who were instructed only to read the questions out loud and ask the respondent to choose between the available answers. The interviewers were explicitly instructed not to engage in the interpretation of the questions with the respondent. The difference is explored more in depth in Rasmussen et al. [13].

The definition of bothersome UI was a constructed by two questions regarding worry related to having UI and the degree of influence on daily activity. It is possible that some respondents have answered negatively on these two questions but still feel bothered by UI.

Including stratified analyses based on gravity of bothersome UI could have added to the results but was not a specific aim of this study.

We included three different types of UI for the survey. UI without stress or urge may include different kinds of UI, e.g. postural incontinence, continuous incontinence, insensible incontinence, and coital incontinence, but the study does not provide any details about this. We did not ask specific about mixed incontinence, rather we explored urge and stress UI separately, but it was possible to report more than one of the symptoms and considerable overlap may have been reported.

In addition to the predefined barriers, the questionnaire comprised an “other” category with the possibility to express other barriers. In total, 27% of the women with bothersome UI reported ‘other’ barriers for healthcare-seeking. Qualitatively exploring the statements in the “other” category is however beyond the scope of this study, but the relative high degree of answers in this category indicates that other barriers than those predefined should be explored in future studies.

### Comparison with existing literature

A Danish study specifically about UI in the general population reached a slightly higher response rate (66.6%) and found a much higher prevalence of 46.4% [3] compared to the total prevalence of 16.4% in the present study. However, the study by Schreiber Pedersen et al. measured UI in general and not only bothersome UI which may explain the substantial difference. Further, it has been shown that the prevalence of UI varies greatly between studies depending on definitions and designs [4]. Therefore, it is recommended to assess the bothersomeness of UI and to use non-UI focused surveys to assess the prevalence of UI in the general population as symptomatic women may be more prone to answer questionnaires leading to an overestimation of the prevalence [4].

Regarding the associations between SES and healthcare-seeking one study found that ethnicity and education were not significantly associated with seeking treatment for UI [19]. However, this study focused only on women during the menopausal transition and consequently only a minor part of the general population of women. In contrast, we included adult women of all ages in our study, which may explain why we found SES to be associated with healthcare-seeking with UI.

## Conclusion

Bothersome UI is common, often not presented to the GP, and younger women are more likely to report barriers for GP contact with UI. Women with lower educational level are more likely to be worried about what the doctor might find and be too embarrassed to contact their GP when being bothered by UI. This knowledge underlines the importance of the GP being aware of identifying women bothered by UI for whom effective treatment options to alleviate the symptoms are available. A special focus should be on women with lower educational level who might be at increased risk of hesitating to contact the GP and on practice organization and structure which may play a role in the way patients and GPs meet in general practice.

## Supplementary Information

**Additional file 1**.

## Data Availability

The datasets are not publicly available due to the data protection regulations of the Danish Data Protection, Statistics Denmark and the Danish Health and Medicines Authority. Access to data is strictly limited to the researchers who have obtained permission for data processing. This permission was giving to the Research Unit of General Practice, Department of Public Health, University of Southern Denmark.

## Abbreviations

UI: Urinary incontinence
GP: General practitioner
SES: Socioeconomic status
OR: Odds ratio
CI: Confidence interval
