# Supplementary material for: Women’s barriers for contacting their general practitioner when bothered by urinary incontinence: a population-based cross-sectional study
Source: BMC Urol. 2021 Jul 12;21:99. doi: 10.1186/s12894-021-00864-x (PMC8273936; doi:10.1186/s12894-021-00864-x)
Supplement: Supplementary file 1 — Additional file 1. [file 12894_2021_864_MOESM1_ESM.docx]

**Table 1.a Reported barriers among women who did not contact the GP with bothersome* stress UI**

|  | **Total** | **Being too embarrassed** | **Wasting the GP's time** | **Worried about what the GP might find** | **Being too busy** | **Other** | **None** |
| --- | --- | --- | --- | --- | --- | --- | --- |
|  | N = (100%) | N (%) | N (%) | N (%) | N (%) | N (%) | N (%) |
|  | 1713(100.0%) | 331(19.3%) | 292(17.0%) | 142(8.3%) | 316(18.4%) | 460(26.9%) | 606(35.40%) |
| **Age** |  |  |  |  |  |  |  |
| 20-39 | 224(13.1%) | 61(18.4%) | 57(19.5%) | 26(18.3%) | 56(17.7%) | 66(14.3%) | 58(9.6%) |
| 40-59 | 811(47.3%) | 172(52.0%) | 139(47.6%) | 72(50.7%) | 186(58.9%) | 234(50.9%) | 235(38.8%) |
| 60-79 | 617(36.0%) | 91(27.5%) | 89(30.5%) | 37(26.1%) | 67(21.2%) | 151(32.8%) | 278(45.9%) |
| 80+ | 61(3.6%) | 7(2.1%) | 7(2.4%) | 7(4.9%) | 7(2.2%) | 9(2.0%) | 35(5.8%) |
| **Marital status:** |  |  |  |  |  |  |  |
| Single | 420(24.5%) | 72(21.8%) | 67(22.9%) | 31(21.8%) | 75(23.7%) | 103(22.4%) | 171(28.2%) |
| Married/Cohabiting | 1293(75.5%) | 259(78.2%) | 225(77.1%) | 111(78.2%) | 241(76.3%) | 357(77.6%) | 435(71.8%) |
| **Educational level:** |  |  |  |  |  |  |  |
| Low (<10 years) | 278(16.2%) | 55(16.6%) | 40(13.7%) | 28(19.7%) | 36(11.4%) | 35(7.6%) | 140(23.1%) |
| Middle (10-14 years) | 922(53.8%) | 183(55.3%) | 170(58.2%) | 84(59.2%) | 176(55.7%) | 216(47.0%) | 330(54.5%) |
| High (>=15 years) | 513(29.9%) | 93(28.1%) | 82(28.1%) | 30(21.1%) | 104(32.9%) | 209(45.4%) | 136(22.4%) |
| **Labor market affiliation:** |  |  |  |  |  |  |  |
| Working | 1022(59.7%) | 219(66.2%) | 187(64.0%) | 86(60.6%) | 255(80.7%) | 310(67.4%) | 284(46.9%) |
| Retirement pension | 480(28.0%) | 71(21.5%) | 65(22.3%) | 34(23.9%) | 40(12.7%) | 101(22.0%) | 242(39.9%) |
| Out of workforce | 99(5.8%) | 26(7.9%) | 23(7.9%) | 13(9.2%) | 14(4.4%) | 20(4.3%) | 33(5.4%) |
| Disability pension | 112(6.5%) | 15(4.5%) | 17(5.8%) | 9(6.3%) | 7(2.2%) | 29(6.3%) | 47(7.8%) |
| **Equivalence weighted disposable income:** |  |  |  |  |  |  |  |
| Low (1^st^ quartile) | 301(17.6%) | 64(19.3%) | 48(16.4%) | 39(27.5%) | 50(15.8%) | 61(13.3%) | 122(20.1%) |
| Middle (2^nd^ and 3^rd^ quartile) | 928(54.2%) | 194(58.6%) | 168(57.5%) | 75(52.8%) | 160(50.6%) | 242(52.6%) | 338(55.8%) |
| High (4^th^ quartile) | 484(28.3%) | 73(22.1%) | 76(26.0%) | 28(19.7%) | 106(33.5%) | 157(34.1%) | 146(24.1%) |
| **Ethnicity:** |  |  |  |  |  |  |  |
| Danish | 1628(95.0%) | 310(93.7%) | 275(94.2%) | 135(95.1%) | 292(92.4%) | 441(95.9%) | 580(95.7%) |
| Immigrants and descendants of immigrants | 85(5.0%) | 21(6.3%) | 17(5.8%) | 7(4.9%) | 24(7.6%) | 19(4.1%) | 26(4.3%) |

*Bothersome UI is defined by being either moderately to extremely concerned and/or moderately to extremely influenced in their daily activities due to UI

**Table 2.a Reported barriers among women who did not contact the GP with** **bothersome* urge UI or UI without stress or urge**

|  | **Total** | **Being too embarrassed** | **Wasting the GP's time** | **Worried about what the GP might find** | **Being too busy** | **Other** | **None** |
| --- | --- | --- | --- | --- | --- | --- | --- |
| **UI** | N = (100%) | N (%) | N (%) | N (%) | N (%) | N (%) | N (%) |
|  | 1069(100.0%) | 204(19.0%) | 181(16.9%) | 109(10.2%) | 166(15.5%) | 294(27.5%) | 414(38.7%) |
| **Age** |  |  |  |  |  |  |  |
| 20-39 | 111(10.4%) | 23(11.3%) | 27(14.9%) | 15(13.8%) | 27(16.3%) | 27(9.2%) | 34(8.2%) |
| 40-59 | 451(42.2%) | 108(52.9%) | 75(41.4%) | 47(43.1%) | 87(52.4%) | 145(49.3%) | 132(31.9%) |
| 60-79 | 444(41.5%) | 65(31.9%) | 69(38.1%) | 43(39.4%) | 45(27.1%) | 109(37.1%) | 215(51.9%) |
| 80+ | 63(5.9%) | 8(3.9%) | 10(5.5%) | 4(3.7%) | 7(4.2%) | 13(4.4%) | 33(8.0%) |
| **Marital status:** |  |  |  |  |  |  |  |
| Single | 315(29.5%) | 49(24.0%) | 42(23.2%) | 30(27.5%) | 50(30.1%) | 93(31.6%) | 127(30.7%) |
| Married/Cohabiting | 754(70.5%) | 155(76.0%) | 139(76.8%) | 79(72.5%) | 116(69.9%) | 201(68.4%) | 287(69.3%) |
| **Educational level:** |  |  |  |  |  |  |  |
| Low (<10 years) | 206(19.3%) | 45(22.1%) | 40(22.1%) | 28(25.7%) | 24(14.5%) | 33(11.2%) | 98(23.7%) |
| Middle (10-14 years) | 561(52.5%) | 107(52.5%) | 103(56.9%) | 59(54.1%) | 88(53.0%) | 139(47.3%) | 224(54.1%) |
| High (>=15 years) | 302(28.3%) | 52(25.5%) | 38(21.0%) | 22(20.2%) | 54(32.5%) | 122(41.5%) | 92(22.2%) |
| **Labor market affiliation:** |  |  |  |  |  |  |  |
| Working | 513(48.0%) | 110(53.9%) | 88(48.6%) | 48(44.0%) | 125(75.3%) | 164(55.8%) | 154(37.2%) |
| Retirement pension | 383(35.8%) | 56(27.5%) | 60(33.1%) | 33(30.3%) | 26(15.7%) | 83(28.2%) | 199(48.1%) |
| Out of workforce | 81(7.6%) | 18(8.8%) | 15(8.3%) | 15(13.8%) | 8(4.8%) | 23(7.8%) | 24(5.8%) |
| Disability pension | 92(8.6%) | 20(9.8%) | 18(9.9%) | 13(11.9%) | 7(4.2%) | 24(8.2%) | 37(8.9%) |
| **Equivalence weighted disposable income:** |  |  |  |  |  |  |  |
| Low (1^st^ quartile) | 225(21.0%) | 46(22.5%) | 39(21.5%) | 31(28.4%) | 31(18.7%) | 44(15.0%) | 99(23.9%) |
| Middle (2^nd^ and 3^rd^ quartile) | 569(53.2%) | 107(52.5%) | 107(59.1%) | 60(55.0%) | 85(51.2%) | 149(50.7%) | 222(53.6%) |
| High (4^th^ quartile) | 275(25.7%) | 51(25.0%) | 35(19.3%) | 18(16.5%) | 50(30.1%) | 101(34.4%) | 93(22.5%) |
| **Ethnicity:** |  |  |  |  |  |  |  |
| Danish | 1017(95.1%) | 191(93.6%) | 169(93.4%) | 101(92.7%) | 154(92.8%) | 280(95.2%) | 398(96.1%) |
| Immigrants and descendants of immigrants | 52(4.9%) | 13(6.4%) | 12(6.6%) | 8(7.3%) | 12(7.2%) | 14(4.8%) | 16(3.9%) |

*Bothersome UI is defined by being either moderately to extremely concerned and/or moderately to extremely influenced in their daily activities due to UI
